# Multivariate prediction of long COVID headache in adolescents using gray matter structural MRI features

**DOI:** 10.3389/fnhum.2023.1202103

**Published:** 2023-06-01

**Authors:** Minhoe Kim, Sunkyung Sim, Jaeseok Yang, Minchul Kim

**Affiliations:** ^1^Department of Computer Convergence Software, Korea University, Sejong, Republic of Korea; ^2^Department of Radiology, Kangnam Sacred Heart Hospital, Hallym University College of Medicine, Seoul, Republic of Korea; ^3^Chonnam National University Medical School, Gwangju, Republic of Korea

**Keywords:** long COVID headache, multivoxel pattern analysis, connectome-based predictive modeling, structural MRI, adolescents

## Abstract

**Objective:**

Headache is among the most frequent symptoms after coronavirus disease 2019 (COVID-19), so-called long COVID syndrome. Although distinct brain changes have been reported in patients with long COVID, such reported brain changes have not been used for predictions and interpretations in a multivariate manner. In this study, we applied machine learning to assess whether individual adolescents with long COVID can be accurately distinguished from those with primary headaches.

**Methods:**

Twenty-three adolescents with long COVID headaches with the persistence of headache for at least 3 months and 23 age- and sex-matched adolescents with primary headaches (migraine, new daily persistent headache, and tension-type headache) were enrolled. Multivoxel pattern analysis (MVPA) was applied for disorder-specific predictions of headache etiology based on individual brain structural MRI. In addition, connectome-based predictive modeling (CPM) was also performed using a structural covariance network.

**Results:**

MVPA correctly classified long COVID patients from primary headache patients, with an area under the curve of 0.73 (accuracy = 63.4%; permutation *p* = 0.001). The discriminating GM patterns exhibited lower classification weights for long COVID in the orbitofrontal and medial temporal lobes. The CPM using the structural covariance network achieved an area under the curve of 0.81 (accuracy = 69.5%; permutation *p* = 0.005). The edges that classified long COVID patients from primary headache were mainly comprising thalamic connections.

**Conclusion:**

The results suggest the potential value of structural MRI-based features for classifying long COVID headaches from primary headaches. The identified features suggest that the distinct gray matter changes in the orbitofrontal and medial temporal lobes occurring after COVID, as well as altered thalamic connectivity, are predictive of headache etiology.

## Introduction

The 2020–2022 period was marked by a severe pandemic due to a novel coronavirus, namely, COVID-19. In addition to respiratory symptoms, headache is one of the most frequent features accompanying COVID-19 and is described by approximately a quarter of patients (Planchuelo-Gómez et al., 2022). Moreover, some people who recover from acute COVID-19 still exhibit a spectrum of symptoms persisting for weeks and even months, so-called long COVID (Tana et al., 2022). Headache is one of the most disabling symptoms of long COVID, however, there is limited knowledge and currently no consensus about the definition of the syndrome known as long COVID (Tana et al., 2022).

Overall, knowledge of long COVID headache is limited; however, interestingly, specific patterns of neuroimaging findings using a mass-univariate approach are continuously being reported in COVID patients. These changes include structural and functional brain changes as well as connectivity alterations (Douaud et al., 2022; Planchuelo-Gómez et al., 2022; Voruz et al., 2022). For example, one of the pioneering study using the UK biobank observed gray matter reduction in patients with COVID-19, specifically in orbitofrontal cortex and parahippocampal gyrus (Douaud et al., 2022). These findings are reported not only in comparison to healthy controls but also against migraineurs (Planchuelo-Gómez et al., 2022).

Despite these neurobiological signs, accurate diagnosis of long COVID headache remains a challenge and based largely on subjective clinical measures, which are often unreliable, with diagnostic variability between clinicians. Headache accompanying COVID has a phenotype that combines the features of tension-type headache and migraine, making it difficult to discriminate (Tana et al., 2022). It would be clinically valuable to identify biomarkers that improve diagnostic discrimination of long COVID headache from primary headache. In addition, it is difficult for a child to accurately describe the type of headache or accompanying symptoms; therefore, diagnosis may be delayed (Kim, 2022).

Recently, multivariate predictive modeling has become a central method for the analysis of neuroscientific data, replacing classical univariate methods (Hebart and Baker, 2018). Specifically, multivariate modeling aims to develop brain models that are tightly coupled with target outcomes using pattern recognition techniques (or “machine learning”) (Woo et al., 2017). In contrast to the mass-univariate approach, which focuses on permitting the inference that altered region R is responsible, conditional on symptom S (i.e., long COVID headache), and assesses the probability P(R| S), a new trend of multivariate modeling has recently emerged to address the reverse inference that symptom S must have occurred given altered region R being related to P(S| R) (Woo et al., 2017).

In this study, we applied a multivariate modeling approach to discriminate long COVID headache from various primary headaches of migraine, daily headache, and tension-type headache. We used structural MRI scans which is routinely available in clinical settings, and is less expensive, and less burdensome to patients compared to other imaging modalities, such as Positron Emission Tomography or functional MRI (Rubin-Falcone et al., 2018). Specifically, we hypothesize that gray matter and connectivity alterations found in previous mass-univariate approach would aid classify long COVID from primary headache.

## Materials and methods

### Participants

This retrospective study was approved by the Institutional Review Board of Kangnam Sacred Heart Hospital (IRB No. HKS 2022-05-026), and informed consent was waived. We retrospectively enrolled 116 consecutive adolescents aged 8–18 years who visited the pediatric headache clinic at the Kangnam Sacred Heart Hospital between May 2022 and December 2022. During the period, the Omicron variant accounted for over 99% of all COVID-19 cases in Korea (Park et al., 2023). We used the patient’s headache profile as well as structural MRI. We did not include a control group of adolescents without headache to focus on our research purpose; to help discriminate the etiology of headache. To date, long COVID headache does not have a specific clinical presentation, therefore the diagnosis is mainly a diagnosis of exclusion (Tana et al., 2022). We used the inclusion criteria for the long COVID headache (CH) patients adapted from previous studies (Planchuelo-Gómez et al., 2022; Tana et al., 2022), as follows: (1) microbiologically confirmed COVID-19 diagnosis based on a real-time reverse transcriptase-polymerase-chain-reaction (RT–PCR) assay using respiratory tract samples or by the presence of anti-SARS-CoV-2 IgM + IgA antibodies, following World Health Organization protocols; (2) new-onset headache presenting during the acute phase of COVID-19, fulfilling criteria for acute headache attributed to systemic viral infection according to International Classification of Headache Disorders, 3rd edition (ICHD-3) (Arnold, 2018); and (3) persistence of headache for at least 3 months after the acute phase of COVID-19. Because we assumed that sufficient long-term deterioration from long COVID is needed to cause a brain morphological change (Rezaeyan et al., 2022), persistence for a 3-month period was selected as a criterion (National Institute for Health and Care Excellence [NICE], 2020). The diagnosis of patients with primary headache (PH) was based on ICHD-3 criteria (Arnold, 2018), and those were consisted of the migraine, new daily persistent headache, tension-type headache and chronic daily headache.

**FIGURE 1:**
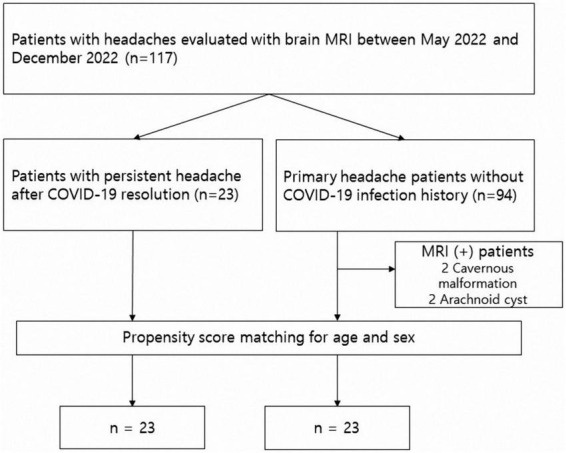
Patient selection procedure for multivariate classification of long COVID headache.

Four participants were excluded for having brain lesions that may affect accurate processing: two for arachnoid cysts and two for cavernous malformations. Then, propensity scores were matched using age and sex as parameters (“*matchit*” R package in R version 4.1.1 (R core team, R foundation for statistical computing, Vienna, Austria) (Stuart et al., 2011; R Core Team, 2013). The propensity score is the estimated probability for each individual in the study to be assigned to the group of interest conditional on all observed confounders. Propensity score matching can effectively adjust for confounders in a retrospective observational study, thus facilitating comparability between patient groups (Baek et al., 2015). Ultimately, 46 age- and sex-matched patients (23 per group) were enrolled. Figure 1 summarizes the patient selection process. For between-group statistical analyses, independent samples *t*-tests were used for continuous variables and Fisher’s exact tests for categorical variables. For statistical tests we used an α-level of 0.05.

### MRI acquisition, quality control, and preprocessing

A Siemens Magnetom Vida 3-Tesla scanner was used to acquire all images. Structural scans of the brain were acquired for each participant using a T1-weighted three-dimensional sagittal magnetization prepared rapid gradient-echo (MP-RAGE) sequence with the following parameters: 176 slices, repetition time = 2020 ms, echo time = 3.05 ms, slice thickness = 0.8 mm, and in-plane resolution of 0.35 × 0.35 mm. Quality checks of images were performed visually and quantitatively with the options “Display slices” and “Check sample homogeneity” in the CAT12 toolbox (Gaser et al., 2022). Moreover, the image “grade” that summarizes measures of image quality by the CAT12 toolbox^1^ was employed to identify images with poor quality and incorrect preprocessing. All of the acquired images achieved a grade of “A-” or above.

Image data preprocessing was conducted using SPM12^2^ and the CAT12 toolbox in the MATLAB 2022b environment.^3^ We performed the preprocessing steps using the CAT12 toolbox with the default settings to improve reproducibility. Briefly, all 3D T1-weighted MRI scans were normalized using an affine function followed by non-linear registration, corrected for bias field in homogeneities, and segmented into gray matter (GM), white matter (WM), and cerebrospinal fluid (CSF) components. We created an age- and sex-specific tissue probability map using TOM8 Toolbox,^4^ following the CAT12 manual (Uusitalo et al., 2021). The Diffeomorphic Anatomic Registration Through Exponentiated Lie Algebra algorithm (DARTEL) was used to normalize the segmented scans into a standard MNI space. Finally, each participant’s modulated and normalized GM tissue segments were smoothed with an 8-mm full width for the half-maximum Gaussian filter.

### Multivoxel pattern analyses

**FIGURE 2:**
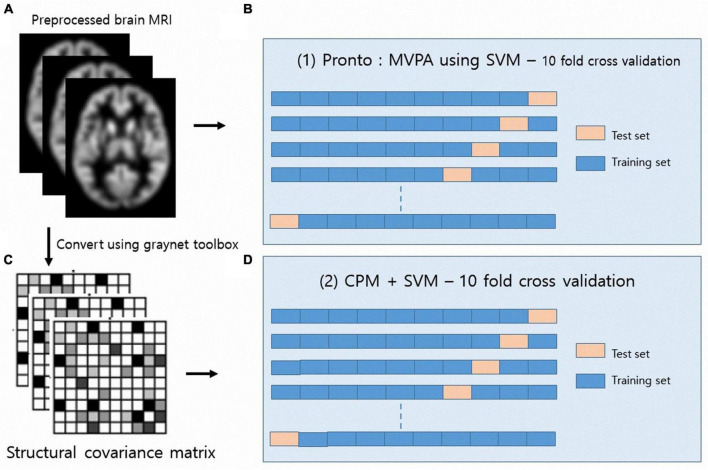
Schematic diagram for multivariate classification of long COVID headache. Two multivariate classifications were conducted using the gray matter volume **(A,B)** and the structural covariance matrix **(C,D)**.

Figure 2 summarizes the two multivariate classifications we conducted. For multivoxel pattern analysis (MVPA), the binary support vector machine (SVM) algorithm was carried out using PRoNTo software [Pattern Recognition for Neuroimaging Toolbox version 3,^5^ (Schrouff et al., 2013)]. According to the manual, PRoNTo uses the linear kernel method to handle the high dimensionality of neuroimaging data. Each preprocessed gray matter image was considered one data point in a high-dimensional space defined by the GM volume (GMV) value. In this high-dimensional space, the linear decision boundaries classify brain scans based on their class label (i.e., the CH and PH groups). We employed 10-fold partitioning of subjects from each group at a time to assess classifier generalizability. Specifically, in each of the 10 cross-validation runs, 10% of the subjects in each group were put aside to test the classification performance, and the remaining 90% of the subjects were used to develop the classifier with all subjects used as testing data at some stage (Figures 2B, D). The SVM finds what is known as the maximum margin decision boundary (Supplementary Figure 1), which is the hyperplane that is furthest from the least discriminating features of the to be discriminated categories, namely the CH and PH groups (Schnyer et al., 2017). When training classifiers using SVM with a linear kernel a default cost parameter of 1 was used. Classification performance was evaluated using the classification accuracy and receiver operating characteristic (ROC) curve analyses derived from probabilistic classifications (Mahmoudi et al., 2012). Accuracy is the total number of correctly classified samples divided by the total number samples. A ROC curve compares the classifier’s true positive rate and false positive rate as the decision threshold varies. The area under the curve (AUC) is thus a summary measure of the performance of the classifier across all decision thresholds, whereby a classifier with perfect classification would achieve an AUC of 1 and a classifier guessing at chance-level an AUC of 0.5 (Lim et al., 2013). A permutation test (permutations = 1000 times) was applied to determine the statistical significance of AUC values (Mahmoudi et al., 2012). Finally, to provide insight into which features drive the classifications, we acquired the average discrimination weight map showing global spatial patterns that best discriminated the group.

### Construction of gray matter structural networks

To construct subjectwise structural networks based on GM volumetric features, we used the open-source python toolbox “graynet” (Raamana and Strother, 2018).^6^ Briefly, 116 cortical and subcortical regions were obtained from the Automated Anatomical Labeling (AAL) atlas (Tzourio-Mazoyer et al., 2002); the modulated, voxelwise GM volumetric distribution (with “mwp1” in CAT12 output) in a given region was converted to a histogram, and the pairwise edge weight was calculated as the histogram correlation between two regions (Tijms et al., 2012; Raamana and Strother, 2017). In this way, a symmetric 116 × 116 structural covariance matrix for each subject was constructed.

### Multivariate classification using structural covariance matrix

To classify CH and PH patients using a structural covariance matrix, we used connectome-based predictive modeling (CPM), an established data-driven protocol for developing predictive models from the brain network (Shen et al., 2017). We modified the CPM by replacing its core learning algorithm with a linear support vector machine (SVM) (Song et al., 2021). The CPM-SVM prediction procedure was done as follows. Across all subjects in the training set, each edge in the structural covariance matrices correlated to the subjects’ group label (i.e., whether each subject was CH or PH) and was considered to be a significant edge if the correlation was below the threshold *p*-value of 0.0005. Next, for each subject, the identified edges were then summed into two predictive variables (i.e., the edges correlating positively and negatively with CH), and the SVM model was trained and tested (Yang et al., 2021). Similar to the MVPA, we used a 10-fold partitioning of subjects from each group at a time and the classification accuracy AUC to evaluate classification performance. A 1000 permutations test was used to determine the significance of our model performance.

For interpretation purposes, we identified edges constantly selected during the cross-validation, namely, “consensus edges.” The edges were visualized using BrainNet Viewer (Xia et al., 2013).

## Results

Table 1 summarizes the characteristics of the study population. There were no significant differences with respect to age or sex in those matched by propensity scoring. The PH patients comprised 7 cases of migraine with aura, 10 cases of migraine without aura, 2 cases of new daily persistent headache and 4 cases of tension-type headache. Among the migraineurs, 15 were episodic migraine while the others were chronic.

**TABLE 1:** Clinical and demographic characteristics of adolescents with long COVID headache and primary headache.

	COVID-19 headache (*n* = 23)	Primary headache (*n* = 23)	Statistical test
Sex (male/female)	12/11	12/11	*p* = 1
Age (years)	11.56 ± 2.66	11.65 ± 2.52	*t* = 0.11, *p* = 0.91
Headache frequency (days/week)	4.86 ± 2.20	4.61 ± 2.48	*t* = 0.37, *p* = 0.71
Total intracranial volume (mm^3^)	1524.8 ± 95.82	1462.3 ± 126.27	*t* = 1.89, *p* = 0.07

Data are the mean ± standard deviation. Independent sample *t*-test for continuous variables and the Fisher’s exact test for categorical variables.

### Multivoxel pattern analyses

**FIGURE 3:**
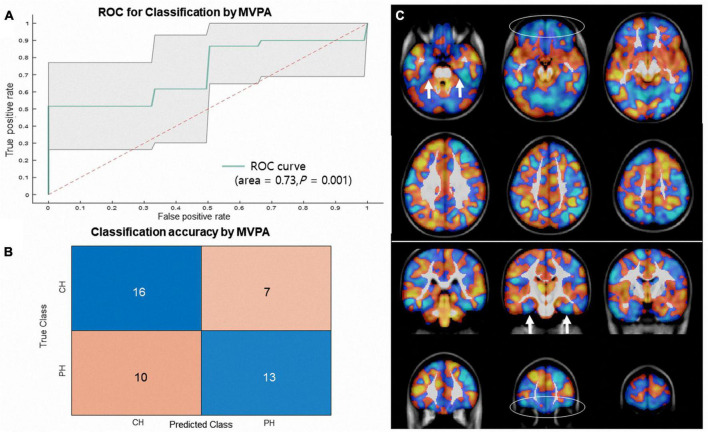
**(A)** Shows receiver operating characteristic (ROC) curves and **(B)** shows confusion matrix generated for classification of long COVID headache from primary headache (cyan line, AUC = 0.73, permutation *p* = 0.001, accuracy = 63.4%; CH class accuracy = 69.5%, PH class accuracy = 56.5%) based on MVPA using SVM. The shaded area represents the 95% confidence interval. **(C)** Shows a non-thresholded multivariate (SVM) weight map overlaid on a T1-weighted MRI image (raw image available at https://neurovault.org/images/795018/). The colors represent relative positive weight distributions (orange) and negative weight distributions (cyan). Note that the gray matter volume decrease in the orbitofrontal (ellipse) and parahippocampal (arrows) areas classifies long COVID headaches.

The SVM classifier trained to discriminate between CH and PH patients achieved an AUC of 0.73 (95% confidence interval = [0.58 0.87], permutation *p* = 0.001, accuracy = 63.4%; CH class accuracy = 69.5%, PH class accuracy = 56.5%, Figure 3 shows the AUC curve and weight map). In MVPA analysis, we are generally asking whether the decoding classification accuracies are significantly greater than what would be expected by chance, and significant classification accuracy suggests sufficient information contained in the input feature (e.g., gray matter pattern) to indicate the subject has long COVID headache (Etzel, 2017; Hebart and Baker, 2018).

### Multivariate classification using structural covariance matrix

**FIGURE 4:**
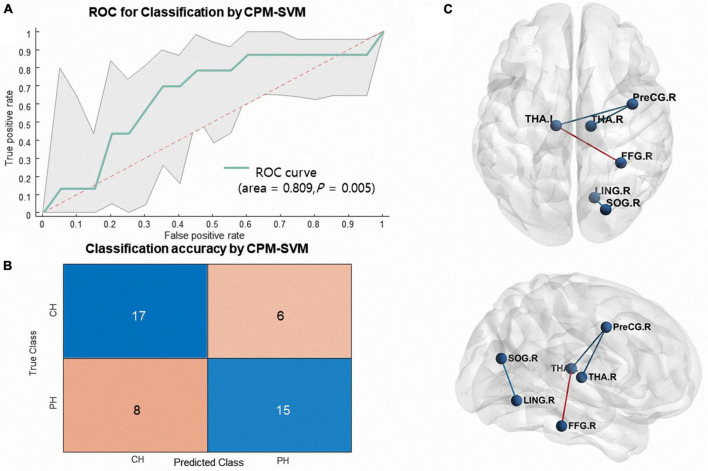
**(A)** Shows receiver operating characteristic (ROC) curves and **(B)** shows confusion matrix generated for classification of long COVID headache from primary headache using a structural covariance matrix (orange line, AUC = 0.81, permutation *p* = 0.005, accuracy = 69.5%; CH class accuracy = 73.9%, PH class accuracy = 65.2%) based on CPM-SVM. The shaded area represents the 95% confidence interval. **(C)** Shows the identified “consensus edges”; the edges colored in red and blue are those increased and decreased in long COVID headache, respectively.

The multivariate classification model using CPM combined with SVM accurately discriminated individuals with CH from PH (AUC = 0.81, 95% confidence interval = [0.68 0.93], permutation *p* = 0.005, accuracy = 69.5%; CH class accuracy = 73.9%, PH class accuracy = 65.2%, Figure 4). There were 4 consensus edges identified during cross-validation: three edges connected the bilateral thalami to brain regions, and one edge connected regions in the right occipital lobe.

## Discussion

In this study, we conducted MVPA and CPM using a structural MRI features to classify PH from CH. We show that it is possible to correctly discriminate adolescents with CH from those with well as structural covariance PH based on gray matter patterns (AUC of 0.73, permutation *p* = 0.001, Figure 3) as matrix (AUC = 0.81, permutation *p* = 0.005, Figure 4).

The multivariate decoding approach can be applied for two main purposes: classifications and interpretation (Tong and Pratte, 2012; Hebart and Baker, 2018). Multivariate decoding for classifications aims to identify biomarkers that can be used to carry out classifications about underlying states, in our case, the etiology of headache. As mentioned above, diagnosis of long COVID headache is not yet standardized. The reported phenotypes of long COVID headaches resemble tension-type headache or migraine-like symptoms, rendering it difficult to discriminate solely based on clinical symptoms (Tana et al., 2022). In addition, one can present with worsening of previously existing headache after COVID. Such cases make it difficult to discriminate the major etiology of headache. Using the unique structural brain alteration pattern and network-wise connectomic alteration, we show that brain images that can be easily acquired may aid the diagnosis.

Overall, knowledge about long COVID headache is accumulating, as well as about treatment. For example, there are ongoing clinical trials examining treatments from sphenopalatine ganglion block to cognitive behavioral therapy (Tana et al., 2022). Accurate diagnosis must precede treatment, and our results show that brain imaging has the potential to aid in diagnosis and tailored treatment. Our study used twenty plus sample size and achieved AUC of 0.73 to 0.81. Those numbers are comparable to previous studies using structural MRI-based MVPA for clinical diagnoses (Lim et al., 2013; Johnston et al., 2015; Rubin-Falcone et al., 2018), however, bigger dataset combined with advanced neuroimaging techniques have potential to improve classification accuracy (Schwedt et al., 2015).

The multivariate linear model weights can provide some insights on which features are driven the classifications (Schrouff and Mourao-Miranda, 2018). The discrimination weight map achieved in MVPA showed negative weight in the bilateral orbitofrontal and medial temporal lobes for CH (Figure 3). It can be interpreted as the gray matter decrease in the orbitofrontal and medial temporal lobes will make SVM classifier move toward (or classifies) CH. Interestingly, the regions with negative weights resembled the results from previous mass-univariate results, that is, involving the orbitofrontal and medial temporal lobes (Díez-Cirarda et al., 2022; Planchuelo-Gómez et al., 2022). For example, Planchuelo-Gómez et al. (2022) reported decreases in gray matter in anterior areas, including the pars orbitalis, the fusiform gyrus and the frontal pole, in patients with persistent headache after COVID-19 resolution. Similar areas have been reported by another large population-based study investigating COVID-19. In a UK Biobank-based study, a greater reduction in gray matter thickness and tissue contrast in the orbitofrontal cortex and parahippocampal gyrus was repeatedly found (Douaud et al., 2022). It is suggested that olfactory cells concentrated in the olfactory epithelium are also particularly vulnerable to coronavirus invasion, and within the olfactory system, direct neuronal connections from the olfactory bulb encompass regions of the piriform cortex (the primary olfactory cortex), parahippocampal gyrus, and orbitofrontal areas (Douaud et al., 2022). Our results indicate that the gray matter in the orbitofrontal lobes and medial temporal lobes is not only decreased but also predictive of long COVID headache. Besides, prominent negative weights were also found in the right middle frontal and parietal cortices (the whole weight map).^7^

With respect to connectivity analysis, the majority of consensus edges connected the bilateral thalami (Figure 4). Although the meaning of structural covariance is rather uncertain, distributional similarities can be used to provide a robust statistical description of individual gray matter morphology and to investigate individual network topological properties (Tijms et al., 2012; Luo et al., 2022). Based on previous studies on the brain, the thalamus is an important sensory relay hub (Cao et al., 2022). In addition, white matter connectivity alterations regarding thalamic radiation have been reported in previous long COVID headache research utilizing diffusion tensor imaging. We suggest that the thalamus plays a role in long COVID headache in sensory connectivity alterations (Planchuelo-Gómez et al., 2022).

There are several study limitations in this study. First, relatively small sample size. Although the number of subjects was comparable with that of similar studies (Lim et al., 2013; Johnston et al., 2015; Rubin-Falcone et al., 2018), replication in a larger study population is desirable. Second, although the AUC of 0.73 to 0.81 passed the permutation test, there would still be a relatively large percentage of incorrectly classified individuals. This may be due to we used simple linear model to retain interpretability. Also, the heterogeneity of the PH patients may account for the modest classification result. Future studies with larger samples may provide better classification accuracy. Third, we did not include a control group of adolescents without headache to focus on our research purpose, that is to help discriminate the etiology of headache. Fourth, about one-third (6 in CH and 7 in PH) of the patients were using acetaminophen on their own, which may or may not be a confounding factor. Fifth, we enrolled patients with persistence of headache for at least 3 months after the acute phase of COVID-19, which may limit the generalizability of the result.

## Conclusion

In conclusion, using only structural MRI, we were able to differentiate adolescents with long COVID headache from those with primary headache. The discrimination pattern and connectivity resembled findings with the mass-univariate approach, supporting that distinct brain gray matter alteration may serve as a biomarker.

## Data availability statement

The raw result of neuroimaging analysis can be seen and downloaded at https://neurovault.org/images/795018/. The de-identified data are available upon reasonable request and subject to approval from the institutional IRB.

## Ethics statement

The studies involving human participants were reviewed and approved by the Institutional Review Board of Kangnam Sacred Heart Hospital (IRB No. HKS 2022-05-026). Written informed consent from the participants’ legal guardian/next of kin was not required to participate in this study in accordance with the national legislation and the institutional requirements.

## Author contributions

MhK: conceptualization, software, investigation, writing, and funding acquisition. SS: conceptualization, investigation, and writing. JY: data curation and writing. McK: conceptualization, software, investigation, writing, funding acquisition, and visualization. All authors contributed to the article and approved the submitted version.

## Footnotes

1
http://www.neuro.uni-jena.de/cat/

2
http://www.fil.ion.ucl.ac.uk/spm/

3
www.mathworks.com

4
https://neuro-jena.github.io/software.html#tom

5
http://www.mlnl.cs.ucl.ac.uk/pronto/

6
https://github.com/raamana/graynet

7
https://neurovault.org/images/795018/

## References

[B1] Arnold M. (2018). Headache classification committee of the international headache society (IHS) the international classification of headache disorders. *Cephalalgia* 38 1–211. 10.1177/0333102417738202 29368949

[B2] Baek S. Park S. H. Won E. Park Y. R. Kim H. J. (2015). Propensity score matching: A conceptual review for radiology researchers. *Korean J. Radiol.* 16 286–296. 10.3348/kjr.2015.16.2.286 25741190 PMC4347264

[B3] Cao Z.-M. Chen Y.-C. Liu G.-Y. Wang X. Shi A.-Q. Xu L.-F. (2022). Abnormalities of thalamic functional connectivity in patients with migraine: A resting-state fMRI study. *Pain Ther.* 11 561–574. 10.1007/s40122-022-00365-1 35220550 PMC9098714

[B4] Díez-Cirarda M. Yus M. Gómez-Ruiz N. Polidura C. Gil-Martínez L. Delgado-Alonso C. (2022). Multimodal neuroimaging in post-COVID syndrome and correlation with cognition. *Brain* 146 2142–2152. 10.1093/brain/awac384 36288544 PMC9620345

[B5] Douaud G. Lee S. Alfaro-Almagro F. Arthofer C. Wang C. Mccarthy P. (2022). SARS-CoV-2 is associated with changes in brain structure in UK Biobank. *Nature* 604 697–707. 35255491 10.1038/s41586-022-04569-5 PMC9046077

[B6] Etzel J. A. (2017). “MVPA significance testing when just above chance, and related properties of permutation tests,” in *Proceedings of the 2017 International Workshop on Pattern Recognition in Neuroimaging (PRNI)*, (Toronto, ON: IEEE), 1–4.

[B7] Gaser C. Dahnke R. Thompson P. M. Kurth F. Luders E. (2022). CAT-a computational anatomy toolbox for the analysis of structural MRI data. *BioRxiv* [Preprint]. 10.1101/2022.06.11.495736 PMC11299546 39102518

[B8] Hebart M. N. Baker C. I. (2018). Deconstructing multivariate decoding for the study of brain function. *Neuroimage* 180 4–18. 10.1016/j.neuroimage.2017.08.005 28782682 PMC5797513

[B9] Johnston B. A. Steele J. D. Tolomeo S. Christmas D. Matthews K. (2015). Structural MRI-based predictions in patients with treatment-refractory depression (TRD). *PLoS One* 10:e0132958. 10.1371/journal.pone.0132958 26186455 PMC4506147

[B10] Kim S. (2022). Pediatric headache: A narrative review. *J. Yeungnam Med. Sci.* 39 278–284. 36102115 10.12701/jyms.2022.00528 PMC9580058

[B11] Lim L. Marquand A. Cubillo A. A. Smith A. B. Chantiluke K. Simmons A. (2013). Disorder-specific predictive classification of adolescents with attention deficit hyperactivity disorder (ADHD) relative to autism using structural magnetic resonance imaging. *PLoS One* 8:e63660. 10.1371/journal.pone.0063660 23696841 PMC3656087

[B12] Luo L. Wen H. Gao L. Li D. (2022). Disrupted gray matter structural networks between active and inactive phases of thyroid-associated ophthalmopathy. *Neuroscience.* 10.21203/rs.3.rs-2279058/v1

[B13] Mahmoudi A. Takerkart S. Regragui F. Boussaoud D. Brovelli A. (2012). Multivoxel pattern analysis for FMRI data: A review. *Comput. Math. Methods Med.* 2012:961257. 10.1155/2012/961257 23401720 PMC3529504

[B14] National Institute for Health and Care Excellence [NICE] (2020). *COVID-19 rapid guideline: Managing the long-term effects of COVID-19.* London: National Institute for Health and Care Excellence (NICE). 33555768

[B15] Park A. K. Kim I.-H. Lee C. Y. Kim J.-A. Lee H. Kim H. M. (2023). Rapid emergence of the omicron variant of severe acute respiratory syndrome coronavirus 2 in Korea. *Ann. Lab. Med.* 43 211–213. 36281518 10.3343/alm.2023.43.2.211 PMC9618911

[B16] Planchuelo-Gómez Ã García-Azorín D. Guerrero ÃL. Rodríguez M. Aja-Fernández S. De Luis-García R. (2022). Structural brain changes in patients with persistent headache after COVID-19 resolution. *J. Neurol.* 270 13–31. 10.1007/s00415-022-11398-z 36178541 PMC9522538

[B17] R Core Team (2013). *R: A language and environment for statistical computing.* Vienna: R Core Team.

[B18] Raamana P. R. Strother S. C. (2017). Histogram-weighted networks for feature extraction, connectivity and advanced analysis in neuroscience. *J. Open Source Softw.* 2:380.

[B19] Raamana P. R. Strother S. C. (2018). Graynet: Single-subject morphometric networks for neuroscience connectivity applications. *J. Open Source Softw.* 3:924.

[B20] Rezaeyan A. Asadi S. Kamrava S. K. Khoei S. Zare-Sadeghi A. (2022). Reorganizing brain structure through olfactory training in post-traumatic smell impairment: An MRI study. *J. Neuroradiol.* 49 333–342. 10.1016/j.neurad.2021.04.035 33957160

[B21] Rubin-Falcone H. Zanderigo F. Thapa-Chhetry B. Lan M. Miller J. M. Sublette M. E. (2018). Pattern recognition of magnetic resonance imaging-based gray matter volume measurements classifies bipolar disorder and major depressive disorder. *J. Affect. Disord.* 227 498–505. 10.1016/j.jad.2017.11.043 29156364 PMC5805651

[B22] Schnyer D. M. Clasen P. C. Gonzalez C. Beevers C. G. (2017). Evaluating the diagnostic utility of applying a machine learning algorithm to diffusion tensor MRI measures in individuals with major depressive disorder. *Psychiatry Res. Neuroimaging* 264 1–9. 10.1016/j.pscychresns.2017.03.003 28388468 PMC5486995

[B23] Schrouff J. Mourao-Miranda J. (2018). “Interpreting weight maps in terms of cognitive or clinical neuroscience: Nonsense?,” in *Proceedings of the 2018 international workshop on pattern recognition in neuroimaging (PRNI)*, (Singapore: IEEE), 1–4.

[B24] Schrouff J. Rosa M. J. Rondina J. M. Marquand A. F. Chu C. Ashburner J. (2013). PRoNTo: Pattern recognition for neuroimaging toolbox. *Neuroinformatics* 11 319–337. 23417655 10.1007/s12021-013-9178-1 PMC3722452

[B25] Schwedt T. J. Chong C. D. Wu T. Gaw N. Fu Y. Li J. (2015). Accurate classification of chronic migraine via brain magnetic resonance imaging. *Headache J. Head Face Pain* 55 762–777. 10.1111/head.12584 26084235 PMC4473808

[B26] Shen X. Finn E. S. Scheinost D. Rosenberg M. D. Chun M. M. Papademetris X. (2017). Using connectome-based predictive modeling to predict individual behavior from brain connectivity. *Nat. Protoc.* 12 506–518. 28182017 10.1038/nprot.2016.178 PMC5526681

[B27] Song K. R. Potenza M. N. Fang X. Y. Gong G. L. Yao Y. W. Wang Z. L. (2021). Resting-state connectome-based support-vector-machine predictive modeling of internet gaming disorder. *Addict. Biol.* 26:e12969. 10.1111/adb.12969 33047425

[B28] Stuart E. A. King G. Imai K. Ho D. (2011). MatchIt: Nonparametric preprocessing for parametric causal inference. *J. Stat. Softw.* 42.

[B29] Tana C. Bentivegna E. Cho S.-J. Harriott A. M. García-Azorín D. Labastida-Ramirez A. (2022). Long COVID headache. *J. Headache Pain* 23 1–12. 35915417 10.1186/s10194-022-01450-8 PMC9340759

[B30] Tijms B. M. Seriès P. Willshaw D. J. Lawrie S. M. (2012). Similarity-based extraction of individual networks from gray matter MRI scans. *Cereb. Cortex* 22 1530–1541. 21878484 10.1093/cercor/bhr221

[B31] Tong F. Pratte M. S. (2012). Decoding patterns of human brain activity. *Annu. Rev. Psychol.* 63 483–509. 21943172 10.1146/annurev-psych-120710-100412 PMC7869795

[B32] Tzourio-Mazoyer N. Landeau B. Papathanassiou D. Crivello F. Etard O. Delcroix N. (2002). Automated anatomical labeling of activations in SPM using a macroscopic anatomical parcellation of the MNI MRI single-subject brain. *Neuroimage* 15 273–289. 11771995 10.1006/nimg.2001.0978

[B33] Uusitalo K. Haataja L. Saunavaara V. Lind A. Vorobyev V. Tilli J. (2021). Performance in hand coordination tasks and concurrent functional MRI findings in 13-year-olds born very preterm. *Pediatric Neurol.* 123 21–29. 10.1016/j.pediatrneurol.2021.07.001 34339952

[B34] Voruz P. Cionca A. Jacot De Alcântara I. NuberChampier A. Allali G. Benzakour L. (2022). Brain functional connectivity alterations associated with neuropsychological performance 6–9 months following SARS-CoV-2 infection. *Hum. Brain Mapp.* 44 1629–1646. 10.1002/hbm.26163 36458984 PMC9878070

[B35] Woo C.-W. Chang L. J. Lindquist M. A. Wager T. D. (2017). Building better biomarkers: Brain models in translational neuroimaging. *Nat. Neurosci.* 20 365–377. 28230847 10.1038/nn.4478 PMC5988350

[B36] Xia M. Wang J. He Y. (2013). BrainNet viewer: A network visualization tool for human brain connectomics. *PLoS One* 8:e68910. 10.1371/journal.pone.0068910 23861951 PMC3701683

[B37] Yang F. N. Hassanzadeh-Behbahani S. Bronshteyn M. Dawson M. Kumar P. Moore D. J. (2021). Connectome-based prediction of global cognitive performance in people with HIV. *Neuroimage Clin.* 30:102677. 10.1016/j.nicl.2021.102677 34215148 PMC8102633

