# Supplementary material for: Multivariate prediction of long COVID headache in adolescents using gray matter structural MRI features
Source: Front Hum Neurosci. 2023 Jun 1;17:1202103. doi: 10.3389/fnhum.2023.1202103 (PMC10267340; doi:10.3389/fnhum.2023.1202103)
Supplement: Supplementary file 1 [file Data_Sheet_1.docx]

Supplementary material

Figure S1. The prediction plot of multivoxel pattern analysis. A prediction plot displays, for a particular fold (y-axis), the output value of the machine’s decision function for each test sample (x-axis, e.g., the function value corresponds to the distance of the test example to the boundary in case of an SVM). The decision boundary/threshold is displayed by a vertical line at the center of the plot.


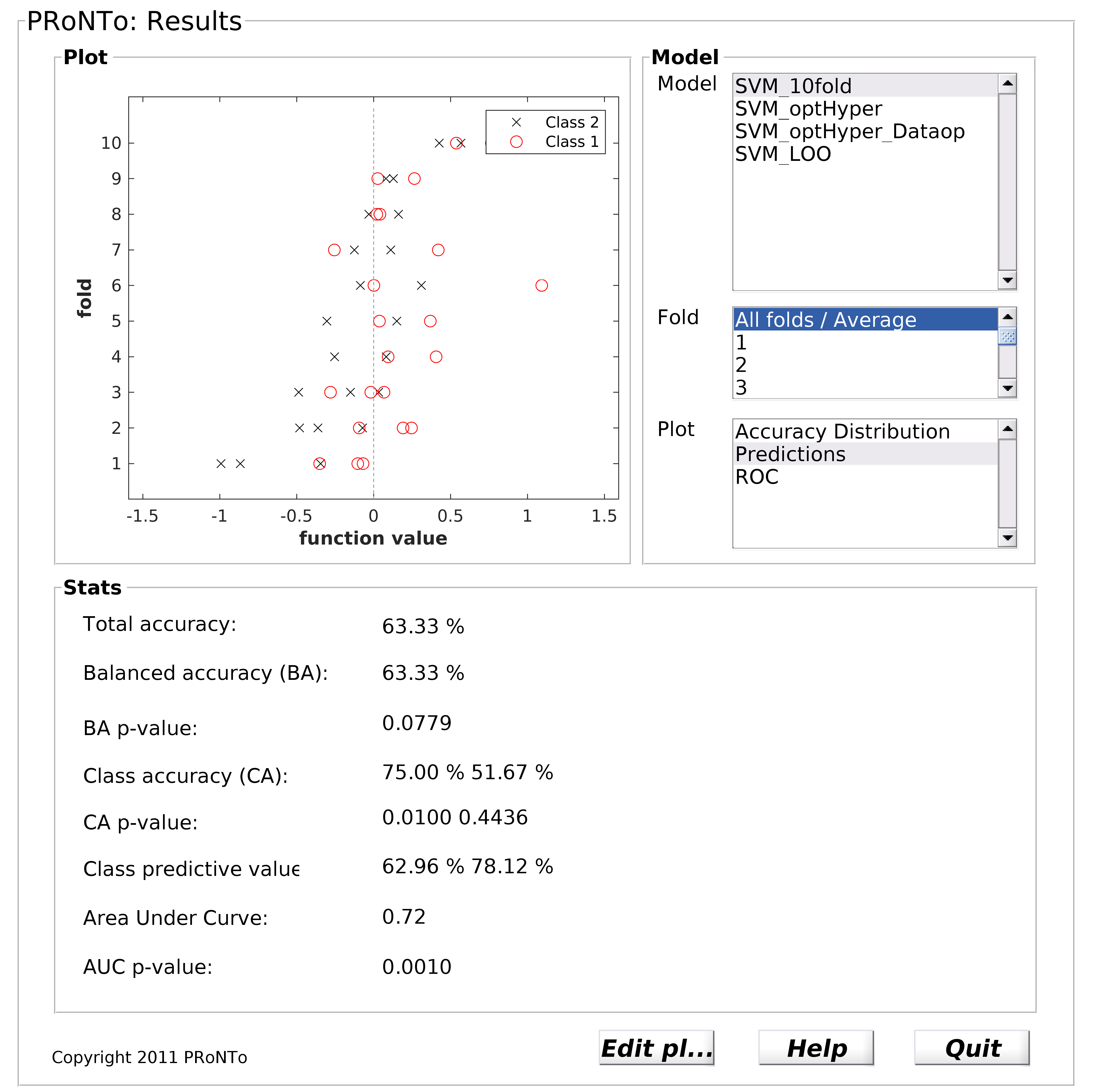


Table S1. Classification performances of multivoxel pattern analysis using other machine learning methods

| Methods | The area under the curve |
| --- | --- |
| SVM with hyper-parameter optimization | 0.72 |
| SVM with hyper-parameter optimization and sample normalization | 0.59 |
| Gaussian process classifier | 0.63 |
